# Adenosine A2A Receptor Contributes to Bidirectional Remodeling of Microglial Inflammatory Responses During Methamphetamine Exposure

**DOI:** 10.3390/ijms27115072

**Published:** 2026-06-04

**Authors:** Zhenping Hou, Xinjie Zhang, Genmeng Yang, Baoyu Shen, Wenjuan Dong, Di Jing, Shijun Hong, Lihua Li

**Affiliations:** National Health Commission (NHC) Key Laboratory of Drug Addiction Medicine, School of Forensic Medicine, Kunming Medical University, 1168 West Chunrong Road, Yuhua Avenue, Chenggong District, Kunming 650500, China; 13783853476@163.com (Z.H.); jir0519@163.com (X.Z.); yanggenmeng@kmmu.edu.cn (G.Y.); baoyu19950629@163.com (B.S.); d1013754851@163.com (W.D.); jingdi2025@163.com (D.J.)

**Keywords:** methamphetamine, adenosine A2A receptor, microglia, neuroinflammation, PKA/PKC signaling pathway

## Abstract

To investigate the role of the adenosine A2A receptor (A2AR) in methamphetamine (MA)-induced microglia-mediated neuroinflammation and to explore the potential signaling mechanism, postmortem human striatal tissue from MA users, a male C57BL/6 mouse model of MA exposure, and the human microglial cell line HMC3 were examined by Western blotting, immunofluorescence, and related assays. Across all three experimental systems, MA exposure significantly upregulated A2AR expression together with alterations in downstream PKA and PKC signaling. These signaling changes were accompanied by parallel upregulation of pro-inflammatory mediators (iNOS, IL-1β, and IL-18) and of anti-inflammatory and repair-associated factors (Arg-1 and IL-10), suggesting that MA did not trigger a simple unidirectional inflammatory program but instead induced multidimensional phenotypic remodeling of microglia that varied with exposure time and dose. Intervention with the selective A2AR antagonist SCH58261 showed that pharmacological inhibition of A2AR markedly attenuated MA-induced alterations in PKA/PKC signaling and suppressed the accompanying shifts in inflammatory mediator expression, thereby mitigating the neuroinflammatory response. These results suggest that A2AR is involved in the modulation of MA-induced microglial inflammatory responses and may contribute to the mixed inflammatory state characterized by simultaneous changes in pro-inflammatory and anti-inflammatory markers, possibly associated with PKA/PKC signaling. This study expands current understanding of the inflammatory basis of MA-related neurotoxicity and suggests A2AR as a potential target for therapeutic intervention in MA abuse.

## 1. Introduction

Drug abuse remains a substantial global health challenge and societal burden. Among stimulants, methamphetamine (MA), commonly known as “ice”, is particularly concerning given its high addictive potential and its potent neurotoxicity within the central nervous system (CNS). Exposure to MA is associated with a broad spectrum of neurological and psychiatric disorders, including cognitive impairment, psychomotor dysfunction, and severe behavioral abnormalities. As a result, the mechanisms and consequences of MA-induced neurotoxicity have remained a central focus of both clinical and experimental research [1,2].

While neuroinflammation is now regarded as a key pathogenic component of MA-induced CNS injury, the precise molecular basis of MA-triggered neuroinflammatory responses has not yet been fully elucidated. As resident immune cells of the CNS, microglia maintain extensive communication with neurons, astrocytes, and other neural cell populations and play a central role in the initiation and progression of neuroinflammation [3,4]. Chronic MA exposure has been shown to induce microglial proliferation and promote the release of pro-inflammatory cytokines and related mediators [2,5], indicating that microglial activation contributes to MA-associated neuroinflammation.

Microglial activation has traditionally been interpreted within the M1/M2 framework, which broadly distinguishes pro-inflammatory conditions from anti-inflammatory states [6]. In this model, M1-like microglia are commonly characterized by inducible nitric oxide synthase (iNOS) and by the production of pro-inflammatory mediators such as tumor necrosis factor-α (TNF-α), interleukin-1β (IL-1β), and interleukin-6 (IL-6), thereby exacerbating neuroinflammatory injury. In contrast, M2-like microglia are typically associated with arginase-1 (Arg-1) and with expression programs linked to anti-inflammatory activity, trophic support, and tissue repair [7,8,9]. Although this framework remains useful for describing broad functional tendencies of microglia during inflammation, it does not fully capture the complex heterogeneity, plasticity, and context dependence of microglial pathological conditions. Furthermore, while growing evidence suggests that MA activates microglia and drives neuroinflammation, the upstream regulatory mechanisms governing this process remain insufficiently defined.

The adenosine A2A receptor (A2AR) is an adenosine-responsive G protein-coupled receptor with broad distribution in human tissues and particularly high abundance in the striatum. In addition to established roles in inflammatory regulation, anti-apoptotic signaling, and immunosuppressive responses [10,11], A2AR has emerged as an important modulator of neuroinflammatory activity, particularly in microglia [12]. Depending on pathological context, A2AR signaling exerts different effects on neuroinflammation with receptor activation suppressing glia-mediated inflammatory responses in multiple sclerosis, chronic cerebral hypoperfusion-induced white matter injury, and spinal cord ischemic injury [13,14,15], whereas A2AR inhibition alleviates lipopolysaccharide-induced neuroinflammation and reduces glutamate release during cerebral ischemic injury, thereby mitigating inflammatory damage [16,17]. Together, these findings indicate that A2AR acts as a context-dependent regulator of inflammatory signaling in the nervous system, with the final biological outcome likely determined by pathological stage, cellular microenvironment, and downstream signaling status. Although previous studies have implicated A2AR in MA use disorder, including suppression of cue-induced MA-seeking behavior after receptor stimulation in rats and reversal of MA-induced deficits in goal-directed behavior [18,19], direct evidence linking A2AR to MA-induced microglial activation and neuroinflammation is still lacking. This is especially relevant in the striatum, which represents both a major site of A2AR enrichment and a region vulnerable to MA-triggered glial activation and neuroinflammatory disruption.

Microglia-mediated neuroinflammation is an important contributor to MA-induced neural injury, and A2AR has been recognized as an important modulator of microglial activation and neuroinflammatory responses [12,20]. The present study investigated the role of A2AR in MA-induced microglial activation and inflammation-related phenotypic remodeling using human brain tissue, an MA-exposed animal model, and in vitro assays. In parallel, the relationship between A2AR and PKA/PKC signaling was evaluated to elucidate the molecular basis of this response and to provide preliminary mechanistic insight into inflammatory regulation in MA-induced neurotoxicity.

## 2. Results

### 2.1. MA Increases A2AR Expression in Human Striatum, Accompanied by Abnormal Expression of Inflammation-Related Molecules

**Figure 1:**
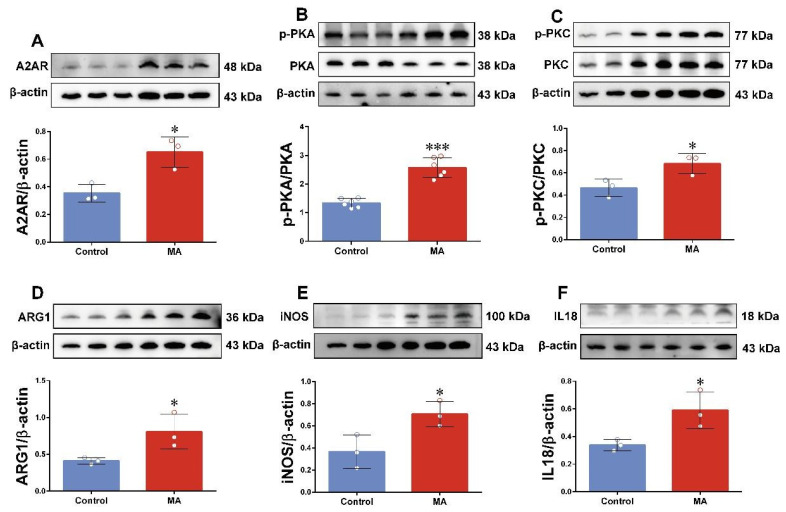
Changes in A2AR expression, PKA/PKC signaling, and inflammatory molecules in striatal tissue from MA abusers. (**A**–**F**) Representative Western blots and quantitative analysis of A2AR (**A**), p-PKA/PKA (**B**), p-PKC/PKC (**C**), Arg-1 (**D**), iNOS (**E**), and IL-18 (**F**) expression in the striatum of MA abusers. * *p* < 0.05, *** *p* < 0.001 vs. control group. Independent-samples *t*-test. All values are presented as mean ± SD (*n* = 3–6).

To characterize A2AR expression and inflammatory changes in striatal tissue from MA abusers, protein expression levels of A2AR, Arg-1, iNOS, and IL-18 were examined in human brain tissue. Compared with the control group, A2AR expression was significantly elevated in the striatum of MA abusers (Figure 1A, *p* < 0.05). Striatal levels of Arg-1 (Figure 1D, *p* < 0.05), iNOS (Figure 1E, *p* < 0.05), and IL-18 (Figure 1F, *p* < 0.05) were also significantly increased, indicating simultaneous elevation of inflammatory and repair-associated molecules. To further assess whether these changes were associated with downstream A2AR signaling, expression and phosphorylation of protein kinase A (PKA) and protein kinase C (PKC) were analyzed. 

Results indicated that phosphorylation of PKA at Thr197 was markedly increased in the striatum of MA abusers compared with controls (Figure 1B, *p* < 0.001), as was phosphorylation of PKC at Thr638 (Figure 1C, *p* < 0.05). Together, these findings indicate that MA exposure is associated with striatal A2AR upregulation, alterations in PKA/PKC signaling, and coordinated changes in inflammation-related molecules (Figure 1).

### 2.2. MA Exposure Increases A2AR Expression and Elicits Inflammation-Related Responses in Mouse Striatum

**Figure 2:**
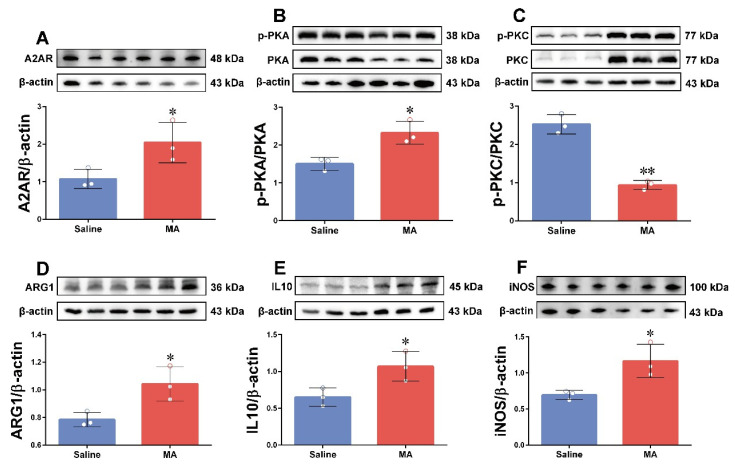
MA exposure increases A2AR expression and induces inflammation-related phenotypic remodeling in the mouse striatum. (**A**–**F**) Western blot analysis of A2AR (**A**), p-PKA/PKA (**B**), p-PKC/PKC (**C**), Arg-1 (**D**), IL-10 (**E**), and iNOS (**F**) expression in the striatum of MA-exposed mice. * *p* < 0.05, ** *p* < 0.01 vs. control group. Independent-samples *t*-test. All values are presented as mean ± SD (*n* = 3).

A mouse model of MA-induced neuroinflammation was established using a previously described exposure model [21] to determine whether the alterations identified in human striatal tissue could be recapitulated in vivo. Striatal A2AR expression, downstream signaling activity, and inflammation-related molecules were then analyzed. Relative to control mice, MA exposure produced a significant increase in A2AR expression in the striatum (Figure 2A, *p* < 0.05). Striatal phosphorylation of PKA at Thr197 was also significantly elevated (Figure 2B, *p* < 0.05), whereas phosphorylation of PKC at Thr638 was significantly reduced (Figure 2C, *p* < 0.01). In parallel, expression levels of Arg-1 (Figure 2D, *p* < 0.05), IL-10 (Figure 2E, *p* < 0.05), and iNOS (Figure 2F, *p* < 0.05) were all significantly increased. Therefore, MA exposure altered the expression of inflammation-related molecules in the mouse striatum and was accompanied by changes in A2AR and downstream signaling. This pattern is broadly consistent with that observed in human brain tissue (Figure 2).

### 2.3. MA Induces Inflammation-Related Phenotypic Remodeling and Alters A2AR Expression in HMC3 Cells in a Time- and Dose-Dependent Manner

**Figure 3:**
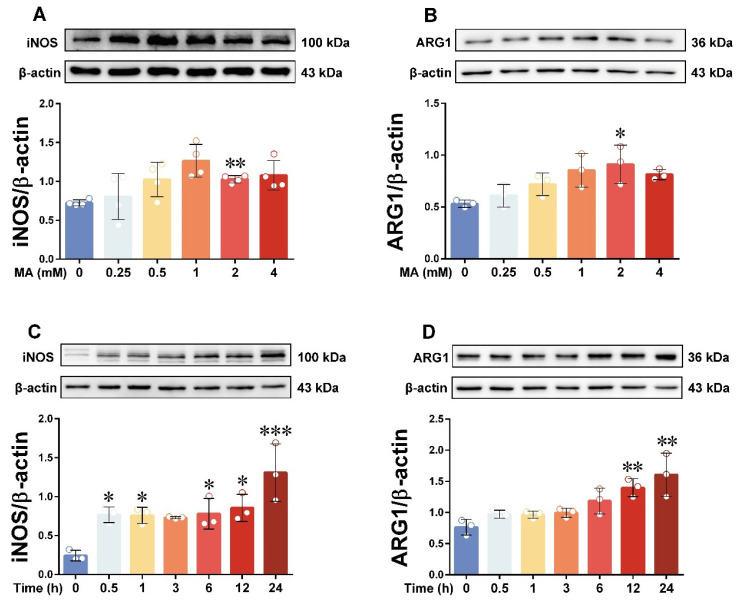
MA exposure induces time- and dose-dependent inflammation-associated phenotypic changes in HMC3 cells. (**A**–**D**) Western blot analysis of iNOS (**A**,**C**) and Arg-1 (**B**,**D**) expression in HMC3 cells exposed to increasing MA concentrations (0, 0.25, 0.5, 1, 2, and 4 mM) (**A**,**B**) or treated with 2 mM MA for different durations (0, 0.5, 1, 3, 6, 12, and 24 h) (**C**,**D**). * *p* < 0.05, ** *p* < 0.01, *** *p* < 0.001 vs. control group. One-way ANOVA followed by Tukey’s post hoc test. All values are presented as mean ± SD (*n* = 3–4).

To define the inflammatory response induced by MA exposure and its relationship to A2AR expression, in vitro experiments were conducted in the human microglial cell line HMC3. MA induced dose-dependent changes in inflammation-associated phenotypic markers, with progressive increases in both iNOS (Figure 3A, *p* < 0.01) and Arg-1 (Figure 3B, *p* < 0.05) as MA concentration increased. A similar temporal pattern was observed after prolonged exposure, with both iNOS (Figure 3C, *p* < 0.001) and Arg-1 (Figure 3D, *p* < 0.01) significantly upregulated over time. These findings suggest that MA treatment does not simply drive microglia toward a single pro-inflammatory or anti-inflammatory state but rather induces a more complex phenotypic remodeling process. Based on these results, 2 mM MA was selected for subsequent experiments (Figure 3).

**Figure 4:**
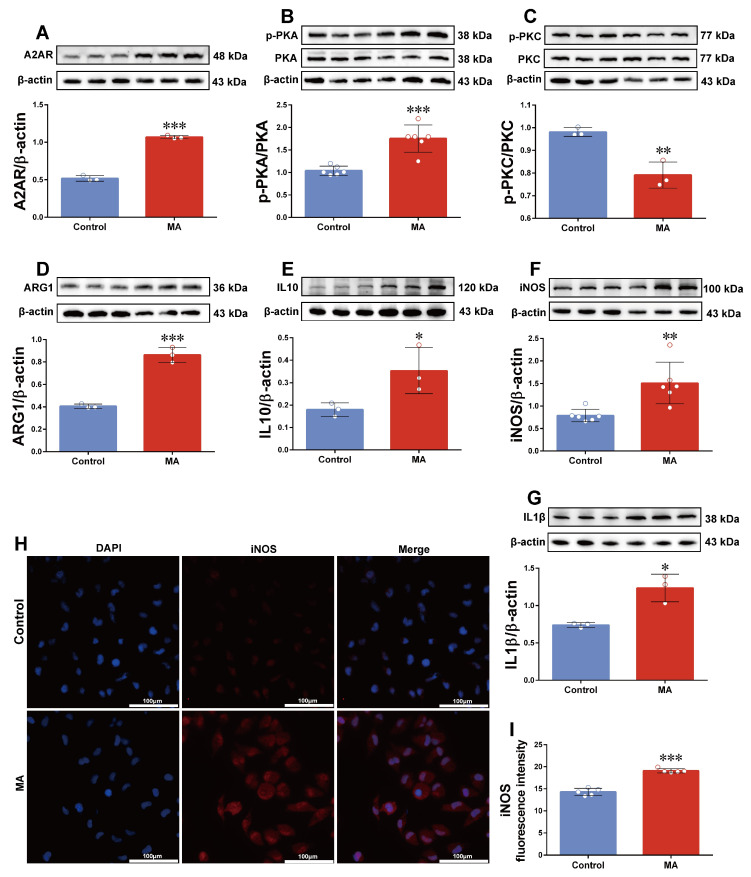
MA exposure at 2 mM for 24 h increases A2AR expression and induces inflammation-related phenotypic remodeling in HMC3 cells. (**A**–**G**) Western blot analysis of A2AR (**A**), p-PKA/PKA (**B**), p-PKC/PKC (**C**), Arg-1 (**D**), IL-10 (**E**), iNOS (**F**), and IL-1β (**G**) expression in HMC3.cells after MA treatment. (**H**,**I**) Immunofluorescence staining and quantification of iNOS expression in HMC3 cells after MA treatment. * *p* < 0.05, ** *p* < 0.01, *** *p* < 0.001 vs. control group. Independent-samples *t*-test. All values are presented as mean ± SD (*n* = 3–6).

After establishing the MA exposure condition that induced inflammation-related phenotypic changes in HMC3 cells, A2AR, downstream signaling molecules, and inflammation-related indicators were further evaluated. Compared with the control group, MA treatment significantly increased A2AR expression (Figure 4A, *p* < 0.01), enhanced phosphorylation of PKA at Thr197 (Figure 4B, *p* < 0.001), and reduced phosphorylation of PKC at Thr638 (Figure 4C, *p* < 0.01). At the same time, expression levels of Arg-1 and IL-10 were significantly elevated (Figure 4D,E, *p* < 0.001 and *p* < 0.05, respectively), while iNOS and the pro-inflammatory cytokine IL-1β were also significantly increased (Figure 4F,G, *p* < 0.01 and *p* < 0.05, respectively). Immunofluorescence analysis further confirmed upregulation of iNOS in HMC3 cells following MA treatment (Figure 4H,I, *p* < 0.001). These findings are broadly consistent with those observed in human and mouse samples, including A2AR upregulation and simultaneous alterations in inflammation-related molecules, although the direction of PKC signaling change was not uniform across systems (Figure 4). 

### 2.4. A2AR Inhibition Attenuates Inflammatory Responses and Modulates PKA/PKC Signaling in the Striatum of MA-Exposed Mice

**Figure 5:**
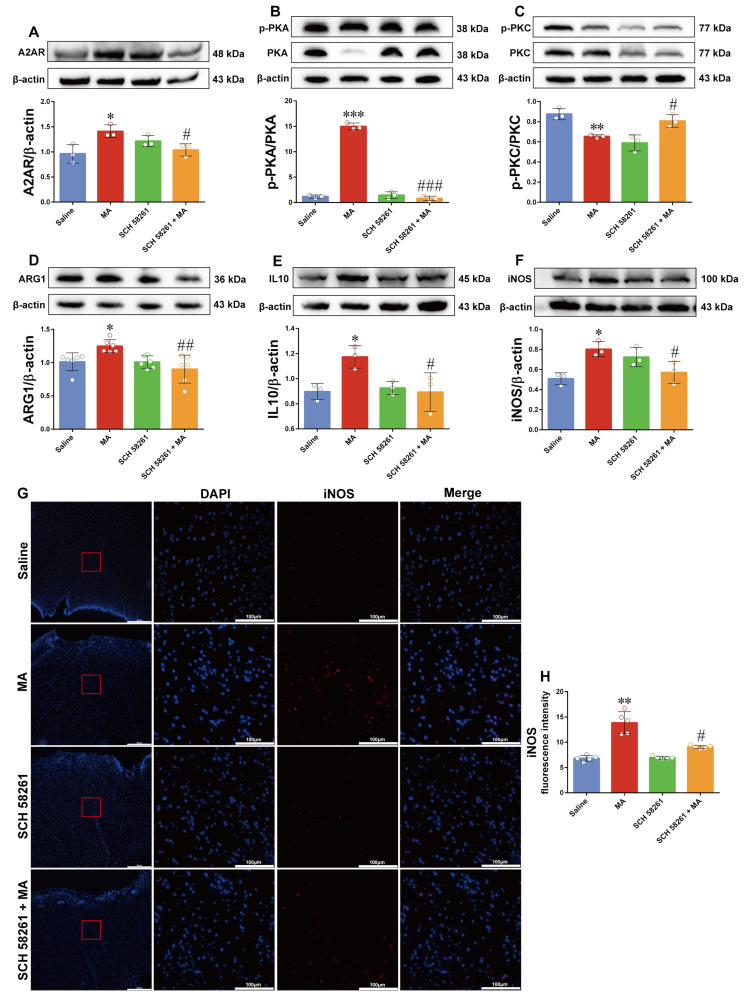
SCH58261-mediated A2AR inhibition reduces changes in A2AR-associated downstream signaling and attenuates MA-induced abnormal inflammatory activation in the striatum of MA-exposed mice. (**A**–**F**) Western blot analysis of A2AR (**A**), p-PKA/PKA (**B**), p-PKC/PKC (**C**), Arg-1 (**D**), IL-10 (**E**), and iNOS (**F**) expression in the mouse striatum after SCH58261 and MA treatment. (**G**,**H**) Immunofluorescence staining and quantification of iNOS expression in the mouse striatum after SCH58261 and MA treatment. * *p* < 0.05, ** *p* < 0.01, *** *p* < 0.001 vs. control group. # *p* <0.05, ## *p* < 0.01, ### *p* < 0.001 vs. MA group. One-way ANOVA followed by Tukey’s post hoc test. All values are presented as mean ± SD (*n* = 3–6).

Consistent increases in A2AR expression and inflammation-related responses were detected in striatal tissue from human MA abusers, as well as in vivo and in vitro models. To determine whether pharmacological inhibition of A2AR modified this response in vivo, the selective A2AR antagonist SCH58261 was administered to MA-exposed mice, followed by assessment of striatal inflammatory markers and A2AR-associated signaling molecules. SCH58261 treatment at 2 mg/kg significantly reduced A2AR expression in the striatum of MA-exposed mice (Figure 5A, *p* < 0.05), lowered phosphorylation of PKA at Thr197 (Figure 5B, *p* < 0.001), and increased phosphorylation of PKC at Thr638 (Figure 5C, *p* < 0.05). In parallel, expression levels of Arg-1, IL-10, and iNOS were markedly reduced (Figure 5D–F, *p* < 0.01, *p* < 0.05, and *p* < 0.05, respectively). Immunofluorescence analysis further showed that SCH58261 decreased iNOS expression in the striatum of MA-exposed mice (Figure 5G,H, *p* < 0.05). Together, these findings suggest that A2AR inhibition does not simply redirect microglia from an M1-like state toward an M2-like state but instead may broadly attenuate the abnormal immune activation program induced by MA exposure (Figure 5).

### 2.5. A2AR Inhibition Attenuates MA-Induced Inflammatory Responses and Modulates PKA/PKC Signaling in HMC3 Cells

**Figure 6:**
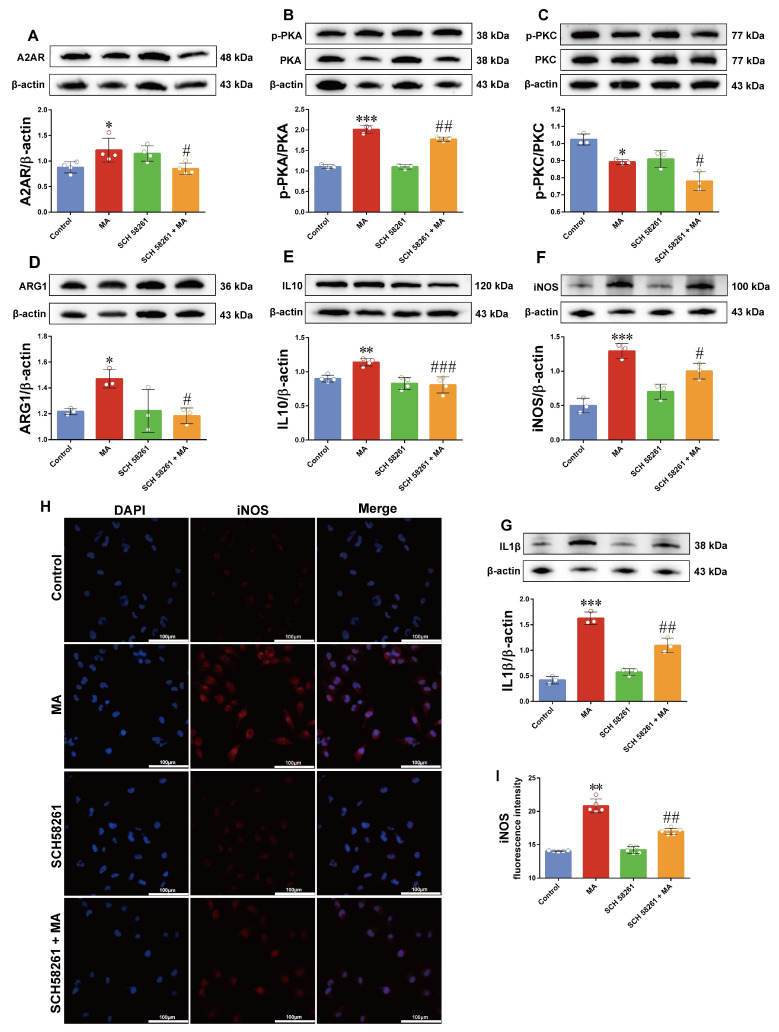
SCH58261-mediated A2AR inhibition reduces A2AR and downstream signaling changes and attenuates MA-induced abnormal inflammatory activation in MA-treated HMC3 cells. (**A**–**G**) Western blot analysis of A2AR (**A**), p-PKA/PKA (**B**), p-PKC/PKC (**C**), Arg-1 (**D**), IL-10 (**E**), iNOS (**F**), and IL-1β (**G**) expression in HMC3 cells after SCH58261 and MA treatment. (**H**,**I**) Immunofluorescence staining of iNOS expression in HMC3 cells after SCH58261 and MA treatment. * *p* < 0.05, ** *p* < 0.01, *** *p* < 0.001 vs. control group. # *p* < 0.05, ## *p* < 0.01, ### *p* <0.001 vs. MA group. One-way ANOVA followed by Tukey’s post hoc test. All values are presented as mean ± SD (*n* = 3–5).

As SCH58261 mitigated MA-induced inflammatory responses in the striatum and was accompanied by changes in PKA/PKC signaling, the effects of A2AR inhibition were further examined in HMC3 cells in vitro. Results showed that SCH58261 treatment significantly reduced A2AR expression in MA-treated HMC3 cells (Figure 6A, *p* < 0.05) and decreased phosphorylation of PKA at Thr197 (Figure 6B, *p* < 0.01) and PKC at Thr638 (Figure 6C, *p* < 0.05). In addition, the expression levels of Arg-1 and the anti-inflammatory cytokine IL-10 were significantly decreased (Figure 6D,E, *p* < 0.05 and *p* < 0.001, respectively), similar to the expression levels of iNOS and the pro-inflammatory cytokine IL-1β (Figure 6F,G, *p* < 0.05 and *p* < 0.01, respectively). Immunofluorescence further confirmed a marked reduction in iNOS expression in MA-treated HMC3 cells after SCH58261 administration (Figure 6H,I, *p* < 0.01), suggesting that A2AR inhibition may broadly reduce the abnormal inflammatory activation program induced by MA (Figure 6).

## 3. Discussion

A2AR is a major adenosinergic signaling receptor in the CNS and is especially enriched in the striatum, where it contributes to neuroinflammatory regulation, synaptic function, and neurodegenerative progression [22,23,24,25]. Within the CNS immune system, microglia represent a major effector cell population through which A2AR may modulate inflammatory activity. Previous studies have linked A2AR to microglial activation, morphological remodeling, and release of inflammatory mediators [26,27]. However, the inflammatory actions of A2AR do not follow a uniform pattern. Instead, its function is highly dependent on pathological context, with receptor activity promoting either anti-inflammatory restraint or pro-inflammatory signaling according to disease stage, local microenvironment, and stimulus characteristics [27,28]. The present study systematically evaluated the role of A2AR in inflammation-associated microglial phenotypic remodeling under MA exposure. Notably, results showed that A2AR was consistently upregulated in MA-exposed human striatal tissue, mouse striatal tissue, and HMC3 cells, accompanied by changes in PKA/PKC signaling and altered expression of multiple inflammation-related molecules. These findings support involvement of A2AR in the abnormal inflammatory network induced by MA.

MA-induced neurotoxicity arises through multiple interacting mechanisms, including neuroinflammation, oxidative stress, and mitochondrial dysfunction [29,30,31]. Among these processes, microglia-mediated neuroinflammation is widely regarded as a major driving force in MA-related brain injury [32]. Microglia exhibit pronounced heterogeneity and adopt distinct activation states in response to different pathological conditions. Although the traditional M1/M2 framework does not fully represent the biological complexity of microglial behavior, it still remains a widely used conceptual model for interpreting broad pro-inflammatory and anti-inflammatory functional tendencies [33,34,35].

Across human striatal specimens, striatal tissue from MA-exposed mice, and MA-treated HMC3 cells, A2AR upregulation emerged as a consistent molecular feature, accompanied by changes in PKA/PKC signaling and altered expression of inflammation-related molecules. In parallel with increases in iNOS, IL-1β, and IL-18, expression of Arg-1 and IL-10 was also elevated. These findings suggest that MA-driven microglial activation cannot be adequately described as a simple shift toward either an M1-like or an M2-like state, but is more consistent with a mixed and dynamically remodeled inflammatory phenotype. Within this framework, increased Arg-1 and IL-10 expression is unlikely to signify establishment of a stable, classically protective M2 program; rather, it more likely reflects a compensatory counter-regulatory response elicited under conditions of sustained pro-inflammatory stress. This interpretation was further supported by pharmacological intervention with the selective A2AR antagonist SCH58261. In both in vivo and in vitro systems, A2AR inhibition attenuated MA-induced A2AR upregulation and reduced expression of canonical pro-inflammatory mediators, including iNOS, IL-1β, and IL-18, as well as Arg-1 and IL-10. These findings indicate that A2AR inhibition broadly attenuates the maladaptive hyperactivation program induced by MA and shifts microglia toward a less reactive state, rather than simply redirecting them between polarized phenotypes. Together, these results suggest that A2AR is involved in MA-induced inflammatory remodeling, accompanied by simultaneous changes in pro-inflammatory and anti-inflammatory markers. This pattern may reflect a mixed or dysregulated inflammatory state rather than a simple phenotypic transition.

Evidence from multiple studies has also suggested that A2AR exerts multidirectional effects during inflammatory regulation and does not operate as a consistently suppressive or pro-inflammatory regulator. In some settings, A2AR activation limits inflammatory injury through immunosuppressive mechanisms, whereas under specific pathological conditions, it contributes to development of the inflammatory cascade. A2AR-dependent adenosine signaling has been identified as a critical component in sustaining inflammasome activity in macrophages [36] and has also been linked to enhanced NLRP3 inflammasome activation through NF-κB signaling, thereby promoting the release of pro-inflammatory cytokines such as IL-1β [37]. Under glutamate-rich conditions, A2AR activation has likewise been shown to participate in microglial inflammasome engagement and inflammatory activation [38]. Beyond these pathways, A2AR has been implicated in divergent inflammatory outputs through ERK1/2, IFN-γ, IL-10, COX-2, PGE2, and NGF signaling [39,40,41,42]. Together, these studies indicate that A2AR serves as a context-dependent regulator of inflammatory responses rather than a receptor with a fixed directional role. Consistent with these observations, MA exposure was accompanied by A2AR upregulation together with simultaneous elevation of canonical pro-inflammatory mediators and anti-inflammatory factors, whereas receptor inhibition reduced both groups of molecules. This response pattern is consistent with the complex regulatory characteristics of A2AR and further suggests that, in MA-related neuroinflammation, A2AR does not appear to function as a purely pro-inflammatory or anti-inflammatory factor, but may participate in maintaining an abnormally amplified inflammatory state.

Downstream kinase signaling may provide a plausible molecular basis for this effect. As a G protein-coupled receptor, A2AR is functionally linked to pathways that include cAMP/PKA and PKC, both of which have established roles in inflammatory regulation. Previous studies have shown that A2AR can reduce inflammatory signaling by engaging the cAMP/PKA/CREB axis and thereby limiting NF-κB activity [43]. Under certain conditions, however, A2AR may also interact with mGluR5, activate PKC-dependent signaling, and promote NF-κB-driven IL-1β production, thereby intensifying inflammatory injury [44]. Additional support for placing PKA and PKC downstream of A2AR comes from neuropathic pain studies in which pharmacological inhibition of these kinases has been shown to reverse the effects of A2AR agonists [45]. Consistent with these observations, the present study showed that MA-induced A2AR upregulation was accompanied by changes in PKA/PKC signaling, and SCH58261 intervention altered both kinase activation and the expression of inflammation-related molecules. These observations suggest the involvement of PKA/PKC signaling in A2AR-associated modulation of MA-induced neuroinflammation. The current findings provide evidence of coordinated changes among A2AR, PKA/PKC, and inflammation-related molecules. However, whether A2AR drives downstream inflammatory reprogramming through PKA/PKC requires more definitive testing with selective agonists, pathway-specific inhibitors, and genetic perturbation strategies.

A notable finding of the present study was the lack of concordance in p-PKC/PKC changes across human, murine, and cellular systems. p-PKC/PKC was elevated in human striatal tissue but reduced in MA-exposed mice and HMC3 cells. In addition, the direction of PKC change following SCH58261 treatment was not consistent between the in vivo and in vitro models. These observations suggest that PKC signaling responds to MA exposure and A2AR regulation in a strongly context-dependent manner. Several factors may underlie these discrepancies. First, the human samples likely reflect a chronic and biologically complex disease state shaped by prolonged exposure, whereas the mouse and cell models more closely represent controlled short-term MA exposure. Second, the in vivo microenvironment involves extensive interactions among neurons, astrocytes, endothelial cells, and immune mediators that cannot be reproduced in a single-cell culture system. Third, PKC comprises a family of kinases with multiple isoforms, each of which may contribute differently to inflammatory signaling and stress adaptation; however, the present analysis did not resolve subtype-specific effects. Therefore, the mechanisms underlying the differential PKC responses observed here require cautious interpretation and further mechanistic clarification.

Several study limitations should be acknowledged. First, although the M1/M2 framework remains useful for describing broad inflammatory tendencies, it does not sufficiently capture the complex heterogeneity of microglial states under MA exposure. Therefore, the present evaluation of pro-inflammatory and anti-inflammatory responses relied primarily on changes in standard markers and should not be regarded as a comprehensive definition of microglial functional status. Second, although HMC3 cells provide a stable and practical platform for mechanistic analyses, they are an immortalized human microglial cell line and may not fully recapitulate the phenotype of primary microglia. Further validation in primary cells or more advanced experimental systems is therefore required. Third, the number of human brain tissue samples was relatively limited, and detailed information on postmortem interval, MA exposure duration and intensity, and other potential confounding factors was not fully available for all cases. These factors may have influenced the observed protein expression patterns and should be considered when interpreting the human data. In addition, because A2AR function was assessed mainly through pharmacological inhibition with SCH58261, genetic approaches are needed to further clarify its role in MA-induced neuroinflammation. Finally, although the present data support involvement of the A2AR/PKA/PKC axis in MA-induced neuroinflammation, the deeper signaling architecture remains to be resolved. Whether this pathway converges on mechanisms involving NF-κB, the NLRP3 inflammasome, or signal integration mediated by A2AR heteromers remains to be further explored.

## 4. Materials and Methods

### 4.1. Drugs

Methamphetamine hydrochloride was legally supplied by the Yunnan Research Institute of Criminal Science and Technology of Public Security (Kunming, China; purity ≥ 95%) and was dissolved in 0.9% saline immediately before use. The selective adenosine A2AR inhibitor SCH58261 (MedChemExpress, Princeton, NJ, USA, HY-19533; purity, 99.71%) was dissolved in dimethyl sulfoxide (DMSO) to generate a stock solution. The final DMSO concentration was matched across all experimental groups, and corresponding vehicle controls were included to exclude confounding effects attributable to the solvent.

### 4.2. Animals

Adult male C57BL/6 mice (6–8 weeks old, 21–30 g, *n* = 48) were purchased from SPF (Beijing, China) Biotechnology Co., Ltd. (license No. SCXK [Jing] 2024-0001). Mice were housed under a 12-h reversed light/dark cycle and controlled environmental conditions, including a relative humidity of 50% ± 10%, a temperature of 22 ± 1 °C, with free access to food and water. All animal experiments were approved by the Animal Ethics Committee of Kunming Medical University (Approval No. Kmmu20220735) and were conducted in strict accordance with the Guide for the Care and Use of Laboratory Animals.

The mouse model was established according to the MA exposure paradigm previously developed by our research group [21]. This paradigm represents a short-term repeated MA exposure model and has been validated to induce neuroinflammatory changes. Mice were randomly assigned to four groups: control, MA, SCH58261, and SCH58261+MA (*n* = 12 per group). Mice in the control group received saline once daily for 5 consecutive days. Mice in the MA group received MA at 5 mg/kg once daily for 5 consecutive days. Mice in the SCH58261 group were treated with SCH58261 at 2 mg/kg once daily for 5 consecutive days according to the same schedule as the control group. Mice in the SCH58261+MA group were pretreated with SCH58261 (2 mg/kg) 30 min before MA administration, followed by MA treatment at 5 mg/kg once daily for 5 consecutive days. Twenty-four hours after the final injection, mice were euthanized by isoflurane (RWD Life Science, Shenzhen, China) and perfused with saline. Striatal tissue was dissected, transferred to 1.5-mL centrifuge tubes, and stored at −80 °C for analysis. For histological preparation, mice were further perfused with 4% paraformaldehyde (Servicebio, Wuhan, China) after saline perfusion, and whole brains were collected and fixed in 4% paraformaldehyde at 4 °C for further processing.

### 4.3. Cell Culture

The immortalized human microglial cell line (HMC3) was obtained from the Cell Bank of the Chinese Academy of Sciences (Shanghai, China). Cells were cultured at 37 °C in a humidified incubator containing 5% CO_2_ in high-glucose Dulbecco’s modified Eagle’s medium (DMEM; VivaCell, Shanghai, China, Cat. No. C3113-0500) supplemented with 10% fetal bovine serum (VivaCell, Cat. No. C04001-050X10) and 50 μg/mL penicillin/streptomycin (VivaCell, Cat. No. C3420-0100). For dose-dependent experiments, HMC3 cells were exposed to MA at concentrations ranging from 0 to 4 mM for 24 h. For time-dependent experiments, cells were treated with 2 mM MA for 0–24 h. In the SCH58261+MA group, cells were pretreated with 50 nM SCH58261 for 30 min before MA administration and then incubated with the indicated concentration of MA.

### 4.4. Human Brain Tissue Samples

Postmortem brain specimens from MA abusers (*n* = 6) and control individuals (*n* = 6) were provided by the Forensic Identification Center of Kunming Medical University, with informed consent obtained from family members of the deceased individuals. Eligibility criteria for the MA abuse group were as follows: (1) male sex; (2) age 18–50 years; (3) detectable MA in blood; (4) confirmed history of MA abuse; and (5) absence of CNS disease or injury. Eligibility criteria for the control group were as follows: (1) male sex; (2) age 18–50 years; (3) death due to cardiovascular disease; (4) no history of drug abuse; and (5) absence of CNS disease or injury. Striatal tissue samples were collected and stored at −80 °C for subsequent protein analysis. All procedures were conducted in accordance with legal and ethical requirements and were approved by the Medical Ethics Committee of Kunming Medical University (Approval No. KMMU2022MEC151). 

### 4.5. Western Blotting

Tissue samples were mixed with an appropriate volume of lysis buffer (Epizyme Biotech, Shanghai, China, PC101), homogenized by ultrasonication, and lysed at 4 °C for 30 min. Total protein concentration was determined using a BCA Protein Assay Kit (Epizyme Biotech, ZJ102). After denaturation, 25 μg of protein per sample was separated by gel electrophoresis (Epizyme Biotech, PG213) and transferred to polyvinylidene fluoride (PVDF) membranes (Sigma-Aldrich, St. Louis, MO, USA, IPVH00010). Membranes were blocked in 5% nonfat milk (Epizyme Biotech, PS112) prepared in Tris-buffered saline and then incubated overnight at 4 °C with the following primary antibodies diluted in blocking solution: A2AR (Abcam, Cambridge, MA, USA, AB3461, 1:1000), p-PKA (phospho-Thr197) (Abcam, ab75991, 1:1000), PKA (Proteintech, Wuhan, China, 67491-1-Ig, 1:1000), p-PKC (phospho-Thr638) (Proteintech, 29123-1-AP, 1:2000), PKC (Proteintech, 66421-1-Ig, 1:2000), Arg-1 (Proteintech, 66129-1-Ig, 1:1000), iNOS (Proteintech, 22226-1-AP, 1:1000), IL-18 (Proteintech, 10663-1-AP, 1:1000), IL-10 (Proteintech, 82191-3-RR, 1:1000), IL-1β (Proteintech, 66737-1-Ig, 1:1000), and β-actin (Proteintech, 66009-1-Ig, 1:5000). Membranes were washed three times with TBST, then incubated for 2 h at room temperature with horseradish peroxidase (HRP)-conjugated goat anti-rabbit secondary antibody (Signalway Antibody, College Park, MD, USA, #L3012, 1:5000) or HRP-conjugated goat anti-mouse secondary antibody (Signalway Antibody, #L3032, 1:10,000) diluted in blocking solution. Following three additional TBST washes, immunoreactive bands were visualized using an ECL Substrate Kit (Biosharp Life Sciences, Hefei, China, BL520B). Signal images were captured using a ChemiDoc MP system (Bio-Rad, Hercules, CA, USA) and analyzed with ImageJ v1.46r.

### 4.6. Immunofluorescence Staining

HMC3 cells and tissue sections were fixed in 4% paraformaldehyde for 10 min, washed three times with phosphate-buffered saline (PBS), permeabilized with 0.2% Triton X-100 (Solarbio Life Sciences, Beijing, China 9036-19-5) for 10 min, and blocked with 20% goat serum (Solarbio Life Sciences, SL038) at room temperature for 1 h. Samples were then incubated overnight at 4 °C with anti-iNOS primary antibody (Proteintech, 22226-1-AP, 1:200). After primary antibody incubation, samples were washed three times with PBS and incubated with Alexa Fluor 594-conjugated goat anti-rabbit secondary antibody (Invitrogen, Carlsbad, CA, USA A-11012, 1:200) at 37 °C for 1.5 h, followed by three additional PBS washes. Nuclei were counterstained with mounting medium containing DAPI. Fluorescence images were captured under a fluorescence microscope (Leica, Wetzlar, Germany), and image acquisition parameters were kept identical across all groups. Five randomly selected fields were analyzed from each sample. Quantification was based on mean fluorescence intensity or percentage of positive area, and fluorescence intensity was measured with ImageJ v1.46r.

### 4.7. Statistical Analysis

All data were analyzed using SPSS v26.0 and are presented as mean ± standard deviation (SD). Comparisons between two groups were performed using the independent-samples *t*-test. Experiments screening MA treatment duration and concentration, as well as experiments involving SCH58261 treatment, were analyzed using one-way analysis of variance (ANOVA) followed by Tukey’s HSD post hoc test. Before application of the *t*-test or ANOVA, normality and homogeneity of variance were assessed using the Shapiro–Wilk and Levene tests, respectively. Data that did not conform to a normal distribution were log-transformed. For data that did not meet the assumption of homogeneity of variance, pairwise comparisons were performed using Tamhane’s T2 test. A *p* value less than 0.05 was considered statistically significant.

## 5. Conclusions

Across human striatal tissue, striatal tissue from MA-exposed mice, and MA-treated HMC3 cells, MA exposure was associated with A2AR upregulation, inflammation-related phenotypic remodeling of microglia, and alterations in PKA/PKC signaling. Pharmacological inhibition of A2AR partially attenuated this abnormal inflammatory activation pattern, supporting a functional role for A2AR in the maintenance of MA-induced neuroinflammation. Rather than acting exclusively along a pro-inflammatory or anti-inflammatory axis, A2AR may be involved in broader inflammatory remodeling under MA exposure, potentially associated with PKA/PKC signaling. These results provide experimental insight into the molecular basis of MA-related neuroinflammation and suggest A2AR as a candidate target for therapeutic intervention.

## Data Availability

The datasets used and/or analyzed in the current study are available from the corresponding author upon reasonable request.
